# Campbell Title Registrations to Date ‐ September 2019

**DOI:** 10.1002/cl2.1055

**Published:** 2019-10-03

1

Details of new titles for systematic reviews or evidence and gap maps that have been accepted by the Editor of a Campbell Coordinating Group are published in each issue of the journal. If you would like to receive a copy of the approved title registration form, please send an email to the Managing Editor of the relevant Coordinating Group.

## 1 BUSINESS AND MANAGEMENT

*Work autonomy and management control as predictors of well‐being and performance*

Jonny Gifford, Rossella Barilli, Niklas Frewel

26 June 2019

*Performance pay and employee health*

Karsten Albæk, Tine Jeppesen, Trine Filges, Bjørn Christian Arleth Viinholt

31 May 2019

*Work autonomy and management control as predictors of well‐being and performance*

Jonny Gifford, Rossella Barilli, Niklas Frewel

19 June 2019

## 2 CRIME AND JUSTICE

*What are the effects of different elements of media on radicalisation outcomes?*

Michael Wolfowicz, Badi Hasisi, David Weisburd

21 June 2019

## 3 EDUCATION

*A systematic review of mobile device use in the primary school classroom and impact on pupil literacy and numeracy attainment*

Claire Dorris, Liam O'Hare, Karen Winter

19 July 2019

## 4 INTERNATIONAL DEVELOPMENT

*Impact of conservation agricultural interventions on farm outcomes among smallholder farmers in sub‐Saharan Africa: A systematic review and meta‐analysis*

Edward N. Mwavu, Onan Mulumba, Paul Mukwaya, Enock Ssekuubwa, Obuku Ekwaro, Moses Ocan

24 July 2019

*A map of development evaluations conducted in Uganda 2000‐2018*

Howard White, Timothy Lubanga, Roland Bless Taremwa, Benjamin Kachero, Caroline Otike, Robert Apunyo, Alison Kinengyere, Francis Rathinam, Zeba Siddiqui, Ekwaro Obuku, Ashrita Saran

12 August 2019

*An evidence and gap map of development evaluations conducted in Kenya 2000‐2018*

Ashrita Saran, Rodney Malesi, Timothy Okech, Virginia Thuku, Evans Mokeni, Margaret Githinji, Bosco Okumu, David Ameyaw, Francis Rathinam, Zeba Siddiqui, Howard White

9 September 2019

*Impact assessment of electronic waste recycling practices*

Salsabil Shaikh, Keith Thomas, Segu Zuhair

30 August 2019

*Mitigating climate change in food consumption and food waste: A systematic map of behavioural interventions*

Lucia A. Reisch, Mark A. Andor, Friederike C. Doebbe, Neal R. Haddaway, Johanna Meier

12 September 2019

*Effects of performance‐based financing interventions on the health system and across health sectors in low‐ and middle‐income countries: An evidence gap map*

Miriam N. Nkangu, Lawrence Mbuagbaw, Loveline Lum, Patrick M. Okwen, Orvill Adams, Christine Mathew, Janet Hatcher Roberts, Sanni Yaya

15 March 2019

*Early detection and intervention for reducing the impact of hearing loss in infancy to late adolescence: an evidence and gap map*

Dhanshree R Gunjawate, Rohit Ravi, Carlie Driscoll

16 July 2019

*Gender equality in the distribution of capabilities and resources: An evidence gap map*

Sujatha Srinivasan, Shreya Jha, R Balagopal, Lakshmi Kumar, Santha Sheela Nair

18 March 2019

*Effects of prenatal flavour exposure on subsequent food preferences and birth outcomes of offspring*

Beyza Nur Ustun, Dilara Ozsoy, Judith Covey, Nadja Reissland

16 May 2019

*Examination and reporting of intersectionality in systematic reviews: capturing multidimensional experiences in public health and nutrition interventions*

Manasee Mishra, Andreea Brabete, Jennifer Petkovic, Kate Ghezzi‐Kopel

13 May 2019

## 5 SOCIAL WELFARE

*Effectiveness of intervention for health promotion of refugees and migrant population: an evidence and gap map*

Baby S Nayak, Sheela Shetty, Tenzin Phagdol, Ashrita Saran, Preethy D'souza

15 September 2019

*Technology‐based, digital and digitally‐delivered interventions addressing all forms of intimate partner and domestic violence (IPDV)*

Chuka Emezue, Tina L. Bloom

23 May 2019

*Adult/child ratio and group size in early childhood education and care to promote the development of children aged 0‐5 years*

Nina Thorup Dalgaard, Anja Bondebjerg, Bjørn Christian Arleth Viinholt, Rasmus Henriksen Klokker, Jens Dietrichson

14 August 2019

*Home care package use for preventing patient decline, death, and entrance into hospital and residential care facilities*

Zorah Hilvert‐Bruce, Dimity Crisp, Zita Hilvert‐Bruce

29 May 2019

*Parent/child attachment interventions to improve parent/child attachment and psychosocial adjustment in foster and adoptive parents and children*

Nina Thorup Dalgaard, Maiken Pontoppidan, Morten Kjær Thomsen, Bjørn Christian Arleth Viinholt, Trine Filges

27 June 2019

*Relationship of emotional regulation on video games dependence*

Jorge R. Saraiva, Cátia M. de Carvalho, Pedro Hubert, Luís Azevedo, Jorge Negreiros, Marta Pinto

28 June 2019

